# A comparison of robust Mendelian randomization methods using summary data

**DOI:** 10.1002/gepi.22295

**Published:** 2020-04-06

**Authors:** Eric A. W. Slob, Stephen Burgess

**Affiliations:** ^1^ Erasmus School of Economics Erasmus University Rotterdam Rotterdam The Netherlands; ^2^ Erasmus University Rotterdam Institute for Behavior and Biology Rotterdam The Netherlands; ^3^ Department of Public Health and Primary Care University of Cambridge Cambridge UK; ^4^ MRC Biostatistics Unit University of Cambridge Cambridge UK

**Keywords:** causal inference, Mendelian randomization, pleiotropy, robust estimation, summary statistics

## Abstract

The number of Mendelian randomization (MR) analyses including large numbers of genetic variants is rapidly increasing. This is due to the proliferation of genome‐wide association studies, and the desire to obtain more precise estimates of causal effects. Since it is unlikely that all genetic variants will be valid instrumental variables, several robust methods have been proposed. We compare nine robust methods for MR based on summary data that can be implemented using standard statistical software. Methods were compared in three ways: by reviewing their theoretical properties, in an extensive simulation study, and in an empirical example. In the simulation study, the best method, judged by mean squared error was the contamination mixture method. This method had well‐controlled Type 1 error rates with up to 50% invalid instruments across a range of scenarios. Other methods performed well according to different metrics. Outlier‐robust methods had the narrowest confidence intervals in the empirical example. With isolated exceptions, all methods performed badly when over 50% of the variants were invalid instruments. Our recommendation for investigators is to perform a variety of robust methods that operate in different ways and rely on different assumptions for valid inferences to assess the reliability of MR analyses.

## 1 INTRODUCTION

Mendelian randomization (MR) uses genetic variants as instrumental variables (IV) to determine whether an observational association between a modifiable exposure (often also called the intermediate variable under study or risk factor) and an outcome is consistent with a causal effect (Davey Smith & Ebrahim, 2003; Smith & Ebrahim, 2004). This approach is less vulnerable to traditional problems of epidemiological studies such as confounding and reverse causality. With the increasing availability of genome‐wide association studies that find robust associations between genetic variants and exposures of interest (Welter et al., 2014; Zheng et al., 2017), the potential of this approach is rapidly evolving. A genetic variant is a valid IV if (a) it is associated with the exposure, (b) it has no direct effect on the outcome, and (c) there are no associations between the variant and any potential confounders.

There has been much discussion on the potentials and limitations of MR, as the IV assumptions cannot be fully tested (Davey Smith & Ebrahim, 2003; Glymour, Tchetgen Tchetgen, & Robins, 2012; VanderWeele, Tchetgen, Cornelis, & Kraft, 2014). Violation of the IV assumptions can lead to invalid conclusions in applied investigations. In practice, the exclusion restriction assumption that the proposed instruments (genetic variants) should not have a direct effect on the outcome of interest is debatable, particularly if the biological roles of the genetic variants are insufficiently understood (Glymour et al., 2012; von Hinke, Smith, Lawlor, Propper, & Windmeijer, 2016).

Some genetic variants are associated with multiple traits (Sivakumaran et al., 2011; Solovieff, Cotsapas, Lee, Purcell, & Smoller, 2013). This is referred to as pleiotropy. There are two types of pleiotropy. Vertical pleiotropy occurs when a variant is directly associated with the exposure and another trait on the same biological pathway. This does not lead to violation of the IV assumptions provided the only causal pathway from the genetic variant to the outcome passes via the exposure. Horizontal pleiotropy occurs when the second trait is on a different biological pathway, and so there may exist different causal pathways from the variant to the outcome. This would violate the exclusion restriction assumption. To solve the problems that arise due to horizontal pleiotropy, several robust methods for MR have been developed that can provide reliable inferences when some genetic variants violate the IV assumptions, or when genetic variants violate the IV assumptions in a particular way. To our knowledge, a comprehensive review and simulation study to compare the statistical performance of these different methods has not been performed.

To focus our simulation study and compare the most relevant robust methods for applied practice, we concentrate on methods that satisfy two criteria. First, the method requires only summary data on estimates (beta‐coefficients and standard errors) of genetic variant–exposure and genetic variant–outcome associations. We exclude methods that require individual participant data (Guo, Kang, TonyCai, & Small, 2018; Jiang et al., 2017; Kang, Zhang, Cai, & Small, 2016; Tchetgen Tchetgen, Sun, & Walter, 2017), and those that require data on additional variants not associated with the exposure (DiPrete, Burik, & Koellinger, 2018; O'Connor & Price, 2018). This is because the sharing of individual participant data is often impractical, so that many empirical researchers only have access to summary data, and for fairness, to ensure that all methods are using the same information to make inferences. Second, the method must be performed using standard statistical software packages. We exclude methods requiring convergence checks that cannot be easily automated for a simulation study (Berzuini, Guo, Burgess, & Bernardinelli, 2018) or are computationally infeasible for large numbers of variants in a reasonable running time (Burgess, Zuber, Gkatzionis, & Foley, 2018).

In this article, we review nine robust methods for MR from a theoretical perspective, and evaluate their performance in a simulation study set in a two‐sample summary data setting. The methods differ in how they estimate a causal effect of the exposure on the outcome, as well as in the assumptions required for consistent estimation. We consider the weighted median, mode‐based estimation (MBE), MR‐Pleiotropy Residual Sum and Outlier (MR‐PRESSO), MR‐Robust, MR‐Lasso, MR‐Egger, contamination mixture, MR‐Mix, and MR‐RAPS methods. Some methods take a summarized measure of the variant‐specific causal estimates as the overall causal effect estimate (weighted median, and MBE), whereas others remove or downweight outliers (MR‐PRESSO, MR‐Lasso, and MR‐Robust), or attempt to model the distribution of the estimates from invalid IVs (MR‐Egger, contamination mixture, MR‐Mix, and MR‐RAPS). We also consider the performance of the methods in an empirical example to evaluate the causal effect of body mass index (BMI) on coronary artery disease risk.

This paper is organized as follows. First, we give an overview of the robust methods and compare their theoretical properties. Then, we introduce the simulation framework and applied example to compare their properties in practice. Finally, we discuss the implications of this study for applied practice.

## 2 METHODS

### 2.1 Modelling assumptions and summary data

**Figure 1:**
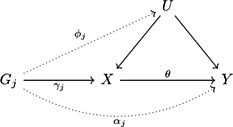
Illustrative diagram showing the model assumed for genetic variant $G_{j}$, with effect $\phi_{j}$ on the unobserved confounder U, effect $\gamma_{j}$ on exposure X, and direct effect $\alpha_{j}$ on outcome Y. The causal effect of the exposure on the outcome is θ. Dotted lines represent possible ways the instrumental variable assumptions could be violated

We consider a model as previously described by Palmer, Thompson, Tobin, Sheehan, and Burton (2008) and Bowden et al. (2017) for J genetic variants $G_{1},G_{2},\text{…},G_{J}$ that are independent in their distributions, a modifiable exposure X, an outcome variable Y, and a confounder U. We assume that all relationships between variables are linear and homogeneous without effect modification, meaning that the same causal effect is estimated by any valid IV (Didelez & Sheehan, 2007). A visual representation of the model is shown in Figure 1.

We assume that summary data are available on genetic associations with the exposure (beta‐coefficient ${\hat{\beta }}_{X_{j}}$ and standard error $\sigma_{X_{j}}$) and with the outcome (beta‐coefficient ${\hat{\beta }}_{Y_{j}}$ and standard error $\sigma_{Y_{j}}$) for each variant $G_{j}$.

### 2.2 Inverse‐variance weighted method

The causal effect of the exposure on the outcome can be estimated using a single genetic variant $G_{j}$ by the ratio method

(1) $${\hat{\theta }}_{R_{j}}=\frac{{\hat{\beta }}_{Y_{j}}}{{\hat{\beta }}_{X_{j}}}.$$

The ratio estimate ${\hat{\theta }}_{R_{j}}$ is a consistent estimate of the causal effect if variant $G_{j}$ satisfies the IV assumptions (Didelez & Sheehan, 2007). If the uncertainty in the genetic association with the exposure is low, then the standard error of the ratio estimate $\sigma_{R_{j}}$ can be approximated as (Thomas, Lawlor, & Thompson, 2007)

(2) $$\sigma_{R_{j}}=\left|\frac{\sigma_{Y_{j}}}{{\hat{\beta }}_{X_{j}}}\right|.$$

The individual ratio estimates can be combined to obtain a single more efficient estimate. The optimally efficient combination of the ratio estimates is referred to as the inverse‐variance weighted (IVW) estimate (Burgess, Butterworth, & Thompson, 2013):

(3) $${\hat{\beta }}_{\text{IVW}}=\frac{\sum_{j=1}^{J}{\hat{\theta }}_{R_{j}}\sigma_{R_{j}}^{-2}}{\sum_{j=1}^{J}\sigma_{R_{j}}^{-2}}=\frac{\sum_{j=1}^{J}{\hat{\beta }}_{X_{j}}{\hat{\beta }}_{Y_{j}}\sigma_{Y_{j}}^{-2}}{\sum_{j=1}^{J}{\hat{\beta }}_{X_{j}}^{2}\sigma_{Y_{j}}^{-2}}.$$

The IVW estimate is equal to the estimate from the two‐stage least squares method that is performed using individual participant data (Burgess, Dudbridge, & Thompson, 2016). It is a weighted mean of the ratio estimates, where the weights are the inverse‐variances of the ratio estimates. The IVW estimate can also be obtained by weighted regression of the genetic associations with the outcome on the genetic associations with the exposure

(4) $${\hat{\beta }}_{Y_{j}}=\theta \;{\hat{\beta }}_{X_{j}}+\varepsilon_{j},\;\varepsilon_{j}∼N\left(0,\sigma_{Y_{j}}^{2}\right).$$

However, the IVW method has a 0% breakdown point, meaning that if only one genetic variant is not a valid IV, then the estimator is typically biased (Bowden, Davey Smith, Haycock, & Burgess, 2016). Bias will be present unless the pleiotropic effects of genetic variants average to zero (balanced pleiotropy) and the pleiotropic effects are independent of the genetic variant–exposure associations (see MR‐Egger method below; Bowden et al., 2017). With the increasing number of variants used in MR investigations, it is increasingly unlikely that all variants are valid IVs. Hence, it is crucial to consider robust estimation methods despite their lower statistical efficiency (i.e., lower power to detect a causal effect).

We proceed to introduce the different robust methods we consider in this study in three categories: consensus methods, outlier‐robust methods, and modelling methods. A summary table comparing the methods is presented as Table 1.

**Table 1:** Summary comparison of methods

Method	Consistency assumption	Strengths and/or weaknesses
Weighted median	Majority valid	Robust to outliers, sensitive to additional/removal of genetic variants, may be less efficient
Mode‐based estimation	Plurality valid	Robust to outliers, sensitive to bandwidth parameter and addition/removal of genetic variants, generally conservative
MR‐PRESSO	Outlier‐robust	Removes outliers, efficient with valid IVs, very high false positive rate with several invalid IVs
MR‐Robust	Outlier‐robust	Downweights outliers, efficient with valid IVs, high false‐positive rate with several invalid IVs
MR‐Lasso	Outlier‐robust	Removes outliers, efficient with valid IVs, high false‐positive rate with several invalid IVs
MR‐Egger	InSIDE	Sensitive to outliers, sensitive to violations of InSIDE assumption, InSIDE assumption often not plausible, may be less efficient
Contamination mixture	Plurality valid	Robust to outliers, sensitive to variance parameter and addition/removal of genetic variants, less conservative than MBE
MR‐Mix	Plurality valid	Robust to outliers, requires large numbers of genetic variants, very high false‐positive rate in several scenarios
MR‐RAPS	Pleiotropic effects (except outliers) normally distributed about zero	Downweights outliers, sensitive to violations of balanced pleiotropy assumption

Abbreviations: InSIDE, Instrument Strength Independent of Direct Effect; IV, instrumental variable; MBE, mode‐based estimation; MR, Mendelian randomization; PRESSO, Pleiotropy Residual Sum and Outlier; RAPS, Robust Adjusted Profile Score.

### 2.3 Consensus methods

A consensus method is one that takes its causal estimate as a summary measure of the distribution of the ratio estimates. The most straightforward consensus method is the median method. Rather than taking a weighted mean of the ratio estimates as in the IVW method, we take the median of the ratio estimates. The median estimator is consistent (i.e., unbiased in large samples) even if up to 50% of the variants are invalid (Bowden et al., 2016). We consider a weighted version of the median method, where the median is taken from a distribution of the ratio estimates in which genetic variants with more precise ratio estimates receive more weight. Here, an unbiased estimate will be obtained if up to 50% of the weight comes from variants that are valid IVs. We refer to this as the “majority valid” assumption.

A related assumption is the “plurality valid” assumption (Guo et al., 2018). In large samples, while ratio estimates for all valid IVs should equal the true causal effect, ratio estimates for invalid IVs will take different values. The “plurality valid” assumption is that, out of all the different values taken by ratio estimates in large samples (we term these the ratio estimands), the true causal effect is the value taken for the largest number of genetic variants (i.e., the modal ratio estimand). For example, the plurality assumption would be satisfied if only 40% of the genetic variants are valid instruments, provided that out of the remaining 60% invalid instruments, no larger group with the same ratio estimand exists. This assumption is also referred to as the Zero Modal Pleiotropy Assumption (ZEMPA; Hartwig, Davey Smith, & Bowden, 2017).

This assumption is exploited by MBE method (Hartwig et al., 2017). As no two ratio estimates will be identical in finite samples, it is not possible to take the mode of the ratio estimates directly. In the MBE method, a normal density is drawn for each genetic variant centered at its ratio estimate. The spread of this density depends on a bandwidth parameter, and (for the weighted version of the MBE method) the precision of the ratio estimate. A smoothed density function is then constructed by summing these normal densities. The maximum of this distribution is the causal estimate.

As these consensus methods take the median or mode of the ratio estimate distribution as the causal estimate, they are naturally robust to outliers, as the median and mode of a distribution are unaffected by the magnitude of extreme values. However, they are still influenced by outliers, as these variants still contribute to determining the location of the median or mode of a distribution. These methods can also be sensitive to changes in the ratio estimates for variants that contribute to the median or mode, and to the addition and removal of variants from the analysis. Additionally, the methods may not be as efficient as those that base their estimates on all the genetic variants.

### 2.4 Outlier‐robust methods

Next, we present three outlier‐robust methods. These methods either downweight or remove genetic variants from the analysis that have outlying ratio estimates. They provide consistent estimates under the same assumptions as the IVW method for the set of genetic variants that are not identified as outliers.

In MR‐PRESSO method (Verbanck, Chen, Neale, & Do, 2018), the IVW method is implemented by regression using all the genetic variants, and the residual sum of squares (RSS) is calculated from the regression equation. The RSS is a heterogeneity measure for the ratio estimates. Then, the IVW method is performed omitting each genetic variant from the analysis in turn. If the RSS decreases substantially compared to a simulated expected distribution, then that variant is removed from the analysis. This procedure is repeated until no further variants are removed from the analysis. The causal estimate is then obtained by the IVW method using the remaining genetic variants.

In MR‐Robust, the IVW method is performed by regression, except that instead of using ordinary least squares regression, MM‐estimation is used combined with Tukey's biweight loss function (Burgess, Bowden, Dudbridge, & Thompson, 2016). MM‐estimation provides robustness against influential points and Tukey's loss function provides robustness against outliers. Tukey's loss function is a truncated quadratic function, meaning that there is a limit in the degree to which an outlier contributes to the analysis (Mosteller & Tukey, 1977). This contrasts with the quadratic loss function used in ordinary least squares regression, which is unbounded, meaning that a single outlier can have an unlimited effect on the IVW estimate.

In MR‐Lasso, the IVW regression model is augmented by adding an intercept term for each genetic variant (Burgess, Bowden, et al., 2016). The IVW estimate is the value of θ that minimizes

(5) $$\sum_{j=1}^{J}\sigma_{Y_{j}}^{-2}{\left({\hat{\beta }}_{Y_{j}}-\theta \;{\hat{\beta }}_{X_{j}}\right)}^{2}.$$

In MR‐Lasso, we minimize

(6) $$\sum_{j=1}^{J}\sigma_{Y_{j}}^{-2}{\left({\hat{\beta }}_{Y_{j}}-\theta_{0j}-\theta \;{\hat{\beta }}_{X_{j}}\right)}^{2}+\lambda \sum_{j=1}^{J}\left|\theta_{0j}\right|,$$

where λ is a tuning parameter. As the regression equation contains more parameters than there are genetic variants, a lasso penalty term is added for identification (Windmeijer, Farbmacher, Davies, & DaveySmith, 2016). The intercept term $\theta_{0j}$ represents the direct (pleiotropic) effect on the outcome, and should be zero for a valid IV, but will be non‐zero for an invalid IV. The causal estimate is then obtained by the IVW method using the genetic variants that had $\theta_{0j}=0$ in Equation (6). A heterogeneity criterion is used to determine the value of λ. Increasing λ means that more of the pleiotropy parameters equal zero and so the corresponding variants are included in the analysis; we increase λ step‐by‐step until one step before there is more heterogeneity in the ratio estimates for variants included in the analysis than expected by chance alone.

The MR‐PRESSO and MR‐Lasso methods remove variants from the analysis, whereas MR‐Robust downweights variants. These methods will be valuable when there is a small number of genetic variants with heterogeneous ratio estimates, as they will be removed from the analysis or heavily downweighted, and so will not influence the overall estimate. In such a case, these methods are likely to be efficient, as they are based on the IVW method. The methods are less likely to be valuable when there is a larger number of genetic variants that are pleiotropic, particularly if the pleiotropic effects are small in magnitude, and when the average pleiotropic effect of non‐outliers is not zero.

### 2.5 Modelling methods

Finally, we present four methods that attempt to model the distribution of estimates from invalid IVs or make a specific assumption about the way in which the IV assumptions are violated. The MR‐Egger method is performed similarly to the IVW method, except that the regression model contains an intercept term $\theta_{0}$:

(7) $${\hat{\beta }}_{Y_{j}}=\theta_{0}+\theta \;{\hat{\beta }}_{X_{j}}+\varepsilon_{j},\;\varepsilon_{j}∼N\left(0,\sigma_{Y_{j}}^{2}\right).$$

This differs from the MR‐Lasso method, as there is only one intercept term, which represents the average pleiotropic effect. The MR‐Egger method gives consistent estimates of the causal effect under the Instrument Strength Independent of Direct Effect (InSIDE) assumption, which states that pleiotropic effects of genetic variants must be uncorrelated with genetic variant–exposure association. As the regression model is no longer symmetric to changes in the signs of the genetic association estimates (which result from switching the reference and effect alleles), we first reorientate the genetic associations before performing the regression by fixing all genetic associations with the exposure to be positive, and correspondingly changing the signs of the genetic associations with the outcome if necessary. The intercept in MR‐Egger also provides a test of the IV assumptions. The intercept will differ from zero when either the average pleiotropic effect is not zero, or the InSIDE assumption is violated. These two conditions (average pleiotropy of zero and InSIDE assumption satisfied) are precisely the conditions required for the IVW estimate to be unbiased.

The contamination mixture method assumes that only some of the genetic variants are valid IVs (Burgess, Foley, Allara, Staley, & Howson, 2020). We construct a likelihood function from the ratio estimates. If a variant is a valid instrument, then its ratio estimate is assumed to be normally distributed about the true causal effect θ with variance $\sigma_{R_{j}}^{2}$. If a variant is not a valid instrument, then its ratio estimate is assumed to be normally distributed about zero with variance $\psi^{2}+\sigma_{R_{j}}^{2}$, where $\psi^{2}$ represents the variance of the estimands from invalid IVs. This parameter is specified by the analyst. We then maximize the likelihood over different values of the causal effect θ and different configurations of valid and invalid IVs. Maximization is performed in linear time by first constructing a profile likelihood as a function of θ, and then maximizing this function with respect to θ. The value of θ that maximizes the profile likelihood is the causal estimate.

The MR‐Mix method (Qi & Chatterjee, 2020) is similar to the contamination mixture method, except that rather than dividing the genetic variants into valid and invalid IVs, the method divides variants into four categories: (a) variants that directly influence the exposure only (valid instruments), and (b) variants that influence the exposure and outcome, (c) that influence the outcome only, and (d) that neither influence the exposure or outcome (invalid instruments). This allows for more flexibility in modelling genetic variants, although potentially leads to more uncertainty in assigning genetic variants to categories.

The MR‐Robust Adjusted Profile Score (RAPS; Zhao, Wang, Bowden, & Small, 2018) method models the pleiotropic effects of genetic variants directly using a random‐effects distribution. The pleiotropic effects are assumed to be normally distributed about zero with unknown variance. Estimates are obtained using a profile‐likelihood function for the causal effect and the variance of the pleiotropic effect distribution. To provide further robustness to outliers, either Tukey's biweight loss function or Huber's loss function (Mosteller & Tukey, 1977) can be used.

Modelling methods are likely to be valuable when the modelling assumptions are correct, but not when the assumptions are incorrect. For example, the MR‐Egger method requires the InSIDE assumption to be satisfied to give a consistent estimate. The MR‐RAPS method is likely to perform well when pleiotropic effects truly are normally distributed about zero, but less well when they are not. The MR‐Mix method is likely to require large numbers of genetic variants to correct classify variants into the different categories. The contamination mixture method is less likely to be affected by modelling assumptions as it does not make such strict assumptions, but it is likely to be sensitive to specification of the variance parameter.

### 2.6 Simulation study

To compare the performance of these methods in a realistic setting, we perform a simulation study. Full details of the simulation study are given in the Supporting Information Material.

For each participant i, we simulate data on J genetic variants $G_{i1},G_{i2},\text{…},G_{iJ}$, a modifiable exposure $X_{i}$, an outcome variable $Y_{i}$, and a confounder $U_{i}$ (assumed unknown). The confounder is a linear function of the genetic variants and an independent error term $\varepsilon_{i}^{U}$. The effect of variant j on the confounder is represented by coefficient $\phi_{j}$ (this is zero for a valid IV). The exposure is linear in the genetic variants, the confounder and an independent error term $\varepsilon_{i}^{X}$. The effect of variant j on the exposure is represented by coefficient $\gamma_{j}$. The outcome is linear in the genetic variants, exposure, confounders, and an independent error term $\varepsilon_{i}^{Y}$. The effect of variant j on the outcome is represented by coefficient $\alpha_{j}$ (again, this is zero for a valid IV). The effect of the exposure on the outcome is represented by θ. The genetic variants are modelled as single nucleotide polymorphisms (SNPs), with a varying minor allele frequency ${\text{maf}}_{j}$, and take values 0, 1, or 2. The minor allele frequencies are drawn from an uniform distribution. The error terms $\varepsilon_{i}^{U}$, $\varepsilon_{i}^{X}$, and $\varepsilon_{i}^{Y}$ each follow an independent normal distribution with mean 0 and unit variance.

We can represent the model mathematically as

(8) $$\begin{array}{ccc} U_{i} & = & \overset{J}{\underset{j=1}{\sum }}\phi_{j}G_{ij}+\varepsilon_{i}^{U},\\ X_{i} & = & \overset{J}{\underset{j=1}{\sum }}\gamma_{j}G_{ij}+U_{i}+\varepsilon_{i}^{X},\\ Y_{i} & = & \overset{J}{\underset{j=1}{\sum }}\alpha_{j}G_{ij}+\theta \;X_{i}+U_{i}+\varepsilon_{i}^{Y},\\ {\text{maf}}_{j} & \sim & U\left(0.1,0.5\right),\\ G_{ij} & \sim & \;\text{Binomial}\;\left(2,{\text{maf}}_{j}\right)\;\;\text{independently}\;,\\ \varepsilon_{i}^{U},\varepsilon_{i}^{X},\varepsilon_{i}^{Y} & \sim & N\left(0,1\right)\;\;\text{independently}\;. \end{array}$$

In brief, we consider three scenarios:

1. balanced pleiotropy, InSIDE satisfied—invalid IVs have direct effects on the outcome generated from a normal distribution centered at zero (for invalid instruments $\alpha_{j}∼N\left(0,0.15\right),\;\phi_{j}=0$);
2. directional pleiotropy, InSIDE satisfied—invalid IVs have direct effects on the outcome generated from a normal distribution centered away from zero (for invalid instruments $\alpha_{j}∼N\left(0.1,0.075\right),\;\phi_{j}=0$);
3. directional pleiotropy, InSIDE violated—invalid IVs have direct effects on the outcome generated from a normal distribution centered away from zero, and indirect effects on the outcome via the confounder (for invalid instruments $\alpha_{j}∼N\left(0.1,0.075\right),\;\phi_{j}∼U\left(0,0.1\right)$).

We simulated data on J=10, 30, and 100 genetic variants. A portion of the genetic variants were invalid IVs (30%, 50%, and 70%), and the direct effects of the variants explain 10% of the variance in the exposure. Summary genetic associations were calculated for the exposure and the outcome on nonoverlapping sets of individuals, each consisting of 10,000 individuals (Haycock et al., 2016). This situation is often referred to as two‐sample summary data MR (Pierce & Burgess, 2013). We considered situations with a null causal effect (θ=0) and a positive causal effect (θ=0.2). In total, 10,000 data sets were generated in each scenario.

Methods can be compared by many metrics, including bias, empirical power, and standard deviation of estimates. We use mean squared error, which is the sum of bias squared plus variance, as the main criterion for comparing methods, as this provides a compromise between bias and precision. However, the relative importance of each metric will depend on the specific features of the application.

### 2.7 Empirical example: The effect of BMI on coronary artery disease (CAD) risk

We also compare the methods in an empirical example considering the effect of BMI on CAD risk. Since BMI is influenced by several biological mechanisms (Monnereau, Vogelezang, Kruithof, Jaddoe, & Felix, 2016), it is likely that the exclusion restriction is not satisfied for all associated genetic variants. Hence it is necessary to use robust methods to analyse these data. Additionally, we consider methods that detect outliers (MR‐Presso, MR‐Robust, MR‐Lasso, contamination mixture, MR‐Mix, and MR‐RAPS), and compare whether the same outliers are detected in each of these methods.

We take 97 genome‐wide significant variants associated with BMI from the GIANT consortium (Locke et al., 2015). Associations with BMI are estimated in up to 339,224 participants from this consortium. Associations with coronary artery disease risk are estimated in up to 60,801 CAD cases and 123,504 controls from the CARDIoGRAMplusC4D Consortium (Nikpay et al., 2015). Association estimates for CAD were available for 94 of these variants.

**Figure 2:**
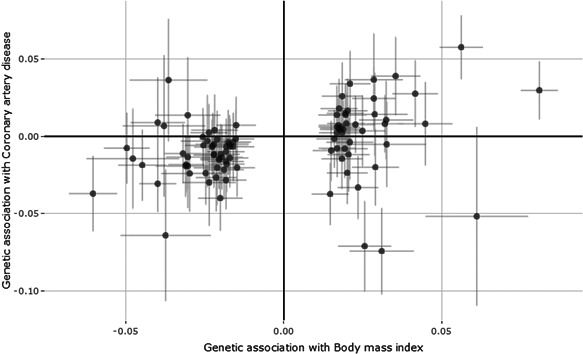
Scatter plot of genetic associations with body mass index (standard deviation units) and coronary artery disease risk (log odds ratios) for 94 variants taken from the GIANT and CARDIoGRAMplusC4D consortia, respectively

The scatter plot of the genetic associations with BMI and CAD risk is shown in Figure 2. While most variants seem to suggest a harmful effect of increased BMI on CAD risk, there is apparent heterogeneity in the IV estimates from each genetic variant individually, as evidenced by Cochran's Q test (Q‐statistic = 235.7, *p* < .001). Even after removing the five outliers as judged by the MR‐PRESSO method, which makes use of the heterogeneity statistic to identify outliers, we still reject the null hypothesis of that the regression model (including an intercept) fits the regression model with no additional variability than would be expected by chance (Q‐statistic = 125.9, *p* = .005). This suggests that some of the variants violate the IV assumptions.

## 3 RESULTS

### 3.1 Simulation study

**Figure 3:**
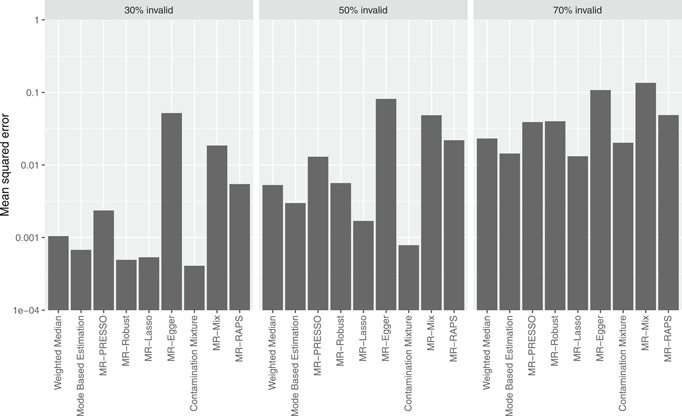
Mean squared errors for the different methods in Scenario 2 (directional pleiotropy, InSIDE satisfied) with a null causal effect for 30 variants. Note the vertical axis is on a logarithmic scale

**Figure 4:**
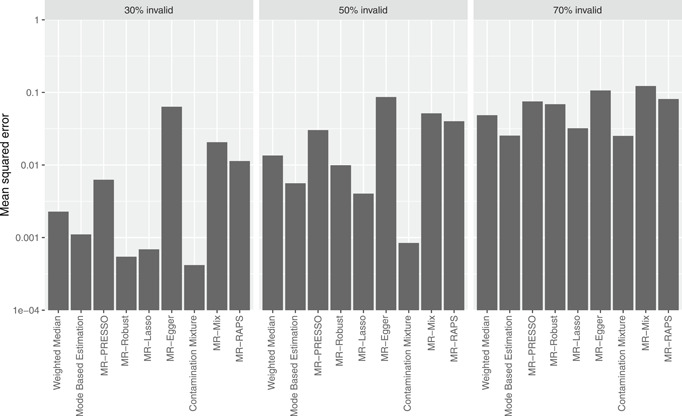
Mean squared errors for the different methods in Scenario 3 (directional pleiotropy, InSIDE violated) with a null causal effect for 30 variants. Note the vertical axis is on a logarithmic scale

The results of the simulation study are presented in Table 2 (10 variants), Table 3 (30 variants), and Table 4 (100 variants). For each scenario, we present the mean, median, and standard deviation of estimates across simulations, and the empirical Type 1 error rate (for a null causal effect) or empirical power (for a positive causal effect) at a 95% confidence level. The empirical Type 1 error rate and empirical power are calculated as the proportion of simulated data sets where zero was not included in the 95% confidence interval. The mean squared error across simulations for the different methods with a null causal effect is presented in Figure 3 (Scenario 2), and Figure 4 (Scenario 3) for 30 variants. The corresponding plots for 10 variants (Figures S1 and S2) and 100 variants (Figures S3 and S4) were broadly similar.

**Table 2:** Mean, median, *SD* of estimates, and Type 1 error/empirical power (%) with 10 genetic variants

	Null casual effect: θ = 0											
	30% invalid				50% invalid				70% invalid			
Method	Mean	Median	*SD*	Type 1 error	Mean	Median	*SD*	Type 1 error	Mean	Median	*SD*	Type 1 error
Scenario 1: Balanced pleiotropy, InSIDE satisfied												
Weighted median	0.000	0.000	0.071	0.139	0.002	0.001	0.132	0.276	0.002	0.000	0.223	0.481
Mode‐based estimation	0.000	0.000	0.101	0.111	0.002	0.000	0.151	0.268	0.002	0.001	0.224	0.619
MR‐PRESSO	0.000	0.000	0.111	0.122	−0.001	0.000	0.178	0.154	0.000	0.001	0.239	0.174
MR‐Robust	0.000	0.000	0.029	0.110	0.001	0.001	0.127	0.076	0.002	0.002	0.224	0.104
MR‐Lasso	0.001	0.000	0.048	0.042	0.000	0.000	0.088	0.076	0.004	0.001	0.183	0.156
MR‐Egger	0.007	0.004	0.419	0.093	0.005	0.008	0.563	0.097	0.006	0.014	0.684	0.098
Contamination mixture	0.000	0.000	0.025	0.052	0.000	0.000	0.077	0.069	0.002	0.000	0.379	0.126
MR‐Mix	0.000	0.000	0.274	0.225	−0.001	0.000	0.431	0.292	0.000	0.000	0.561	0.356
MR‐RAPS	0.000	−0.001	0.106	0.039	0.001	0.000	0.172	0.062	0.001	0.000	0.226	0.083
Scenario 2: Directional pleiotropy, InSIDE satisfied												
Weighted median	0.013	0.006	0.060	0.140	0.036	0.016	0.108	0.287	0.084	0.036	0.175	0.500
Mode‐based estimation	0.007	0.001	0.081	0.114	0.020	0.006	0.122	0.264	0.059	0.030	0.180	0.585
MR‐PRESSO	0.028	0.013	0.079	0.132	0.069	0.031	0.133	0.168	0.122	0.071	0.182	0.214
MR‐Robust	0.003	0.002	0.031	0.106	0.042	0.023	0.105	0.084	0.115	0.094	0.169	0.152
MR‐Lasso	0.008	0.005	0.044	0.056	0.024	0.012	0.082	0.125	0.075	0.035	0.161	0.283
MR‐Egger	0.001	−0.006	0.329	0.093	0.000	−0.013	0.408	0.091	−0.005	−0.012	0.477	0.095
Contamination mixture	0.000	0.001	0.025	0.059	0.003	0.001	0.056	0.078	0.060	0.006	0.281	0.137
MR‐Mix	0.045	0.016	0.200	0.247	0.084	0.023	0.301	0.331	0.144	0.050	0.399	0.443
MR‐RAPS	0.039	0.030	0.082	0.053	0.081	0.071	0.128	0.095	0.130	0.119	0.165	0.152
Scenario 3: Directional pleiotropy, InSIDE violated								
Weighted median	0.022	0.011	0.071	0.179	0.073	0.030	0.137	0.384	0.135	0.080	0.188	0.599
Mode‐based estimation	0.013	0.002	0.090	0.132	0.044	0.011	0.148	0.317	0.094	0.051	0.192	0.621
MR‐PRESSO	0.047	0.023	0.095	0.155	0.113	0.063	0.153	0.223	0.179	0.147	0.185	0.301
MR‐Robust	0.004	0.002	0.032	0.106	0.069	0.040	0.121	0.109	0.169	0.152	0.171	0.216
MR‐Lasso	0.013	0.008	0.050	0.073	0.050	0.024	0.108	0.203	0.122	0.067	0.180	0.415
MR‐Egger	0.049	0.024	0.326	0.098	0.066	0.042	0.411	0.097	0.048	0.034	0.464	0.096
Contamination mixture	0.000	0.000	0.025	0.060	0.005	0.001	0.061	0.080	0.079	0.009	0.273	0.163
MR‐Mix	0.064	0.026	0.207	0.283	0.125	0.040	0.304	0.375	0.196	0.080	0.391	0.529
MR‐RAPS	0.062	0.050	0.091	0.085	0.132	0.118	0.132	0.182	0.188	0.180	0.160	0.262

	Positive causal effect: θ=+0.2											
	30% invalid				50% invalid				70% invalid			
Method	Mean	Median	*SD*	Power	Mean	Median	*SD*	Power	Mean	Median	*SD*	Power
Scenario 1: Balanced pleiotropy, InSIDE satisfied												
Weighted median	0.201	0.200	0.069	0.979	0.201	0.200	0.131	0.939	0.200	0.201	0.221	0.877
Mode‐based estimation	0.198	0.200	0.102	0.983	0.192	0.199	0.156	0.945	0.183	0.193	0.235	0.867
MR‐PRESSO	0.199	0.200	0.106	0.860	0.202	0.201	0.166	0.734	0.200	0.202	0.232	0.564
MR‐Robust	0.200	0.200	0.033	0.953	0.201	0.201	0.129	0.506	0.199	0.200	0.225	0.282
MR‐Lasso	0.200	0.200	0.052	0.962	0.201	0.201	0.091	0.906	0.198	0.200	0.189	0.774
MR‐Egger	0.199	0.201	0.442	0.166	0.199	0.199	0.549	0.122	0.197	0.193	0.660	0.113
Contamination mixture	0.200	0.200	0.028	0.997	0.202	0.201	0.074	0.959	0.228	0.204	0.399	0.704
MR‐Mix	0.210	0.203	0.242	0.562	0.219	0.205	0.370	0.612	0.224	0.210	0.522	0.644
MR‐RAPS	0.200	0.200	0.108	0.538	0.201	0.202	0.168	0.309	0.197	0.201	0.228	0.222
Scenario 2: Directional pleiotropy, InSIDE satisfied												
Weighted median	0.214	0.207	0.060	0.991	0.240	0.216	0.114	0.978	0.285	0.242	0.175	0.952
Mode‐based estimation	0.205	0.201	0.081	0.983	0.219	0.204	0.129	0.961	0.248	0.226	0.180	0.917
MR‐PRESSO	0.225	0.213	0.072	0.945	0.267	0.232	0.129	0.849	0.319	0.274	0.177	0.729
MR‐Robust	0.204	0.203	0.034	0.954	0.244	0.225	0.109	0.646	0.315	0.301	0.168	0.555
MR‐Lasso	0.209	0.206	0.047	0.985	0.225	0.213	0.085	0.971	0.274	0.239	0.161	0.926
MR‐Egger	0.200	0.188	0.323	0.215	0.199	0.187	0.407	0.153	0.196	0.187	0.462	0.133
Contamination mixture	0.201	0.201	0.030	0.997	0.206	0.201	0.085	0.968	0.286	0.210	0.307	0.823
MR‐Mix	0.252	0.228	0.175	0.613	0.291	0.240	0.265	0.664	0.353	0.276	0.367	0.738
MR‐RAPS	0.238	0.229	0.080	0.825	0.285	0.275	0.127	0.675	0.329	0.322	0.164	0.595
Scenario 3: Directional pleiotropy, InSIDE violated								
Weighted median	0.225	0.212	0.074	0.994	0.272	0.233	0.137	0.985	0.339	0.287	0.185	0.975
Mode‐based estimation	0.211	0.201	0.092	0.983	0.239	0.211	0.147	0.961	0.290	0.252	0.189	0.940
MR‐PRESSO	0.243	0.223	0.086	0.925	0.307	0.262	0.149	0.835	0.379	0.342	0.182	0.759
MR‐Robust	0.205	0.204	0.036	0.945	0.271	0.244	0.122	0.651	0.372	0.353	0.168	0.651
MR‐Lasso	0.216	0.210	0.053	0.991	0.250	0.226	0.109	0.981	0.326	0.274	0.179	0.964
MR‐Egger	0.248	0.225	0.330	0.245	0.266	0.242	0.408	0.183	0.251	0.236	0.458	0.155
Contamination mixture	0.201	0.201	0.029	0.996	0.209	0.202	0.082	0.970	0.317	0.217	0.318	0.850
MR‐Mix	0.274	0.240	0.180	0.654	0.327	0.260	0.260	0.713	0.405	0.319	0.357	0.788
MR‐RAPS	0.263	0.251	0.090	0.872	0.329	0.316	0.134	0.797	0.389	0.378	0.158	0.759

Abbreviations: InSIDE, Instrument Strength Independent of Direct Effect; MR, Mendelian randomization; PRESSO, Pleiotropy Residual Sum and Outlier; RAPS, Robust Adjusted Profile Score; SD, standard deviation.

**Table 3:** Mean, median, *SD* of estimates, and Type 1 error/empirical power (%) with 30 genetic variants

	Null casual effect: *θ* = 0											
	30% invalid				50% invalid				70% invalid			
Method	Mean	Median	*SD*	Type 1 error	Mean	Median	*SD*	Type 1 error	Mean	Median	*SD*	Type 1 error
Scenario 1: Balanced pleiotropy, InSIDE satisfied												
Weighted median	0.000	0.000	0.033	0.085	−0.001	0.000	0.066	0.168	−0.002	−0.002	0.134	0.333
Mode‐based estimation	0.000	0.000	0.029	0.052	0.000	0.000	0.063	0.127	0.000	−0.001	0.136	0.494
MR‐PRESSO	0.000	0.000	0.052	0.208	−0.001	0.000	0.091	0.276	−0.002	0.000	0.145	0.351
MR‐Robust	0.000	0.000	0.023	0.069	0.000	0.000	0.075	0.024	−0.001	−0.004	0.172	0.054
MR‐Lasso	0.000	−0.001	0.025	0.038	0.000	0.000	0.036	0.061	−0.001	0.000	0.081	0.111
MR‐Egger	0.004	0.003	0.319	0.068	0.006	0.002	0.400	0.073	−0.010	−0.008	0.464	0.074
Contamination mixture	0.000	0.000	0.022	0.062	0.000	0.000	0.030	0.078	−0.002	0.001	0.177	0.127
MR‐Mix	0.000	0.000	0.141	0.052	0.000	0.000	0.215	0.053	0.002	0.000	0.321	0.036
MR‐RAPS	−0.001	−0.001	0.077	0.019	0.000	−0.003	0.132	0.041	−0.002	−0.004	0.178	0.055
Scenario 2: Directional pleiotropy, InSIDE satisfied												
Weighted median	0.011	0.009	0.031	0.100	0.031	0.021	0.066	0.235	0.083	0.048	0.127	0.438
Mode‐based estimation	0.001	0.000	0.026	0.049	0.006	0.003	0.054	0.132	0.040	0.026	0.113	0.454
MR‐PRESSO	0.024	0.016	0.042	0.230	0.071	0.047	0.089	0.424	0.145	0.119	0.134	0.584
MR‐Robust	0.003	0.002	0.022	0.065	0.034	0.026	0.067	0.030	0.149	0.140	0.133	0.159
MR‐Lasso	0.004	0.003	0.023	0.058	0.014	0.011	0.039	0.135	0.061	0.039	0.097	0.340
MR‐Egger	0.004	−0.004	0.228	0.073	0.001	−0.005	0.285	0.074	−0.002	−0.008	0.328	0.071
Contamination mixture	0.001	0.001	0.020	0.064	0.001	0.001	0.028	0.085	0.015	0.003	0.141	0.140
MR‐Mix	0.018	0.006	0.135	0.078	0.041	0.010	0.216	0.107	0.096	0.010	0.355	0.119
MR‐RAPS	0.046	0.042	0.058	0.051	0.110	0.105	0.099	0.160	0.179	0.175	0.129	0.273
Scenario 3: Directional pleiotropy, InSIDE violated								
Weighted median	0.022	0.017	0.042	0.168	0.067	0.040	0.095	0.401	0.156	0.114	0.155	0.668
Mode‐based estimation	0.002	0.001	0.033	0.057	0.016	0.006	0.073	0.172	0.077	0.048	0.140	0.531
MR‐PRESSO	0.050	0.035	0.061	0.397	0.132	0.108	0.114	0.653	0.232	0.216	0.146	0.816
MR‐Robust	0.004	0.004	0.023	0.052	0.059	0.045	0.080	0.041	0.224	0.216	0.136	0.335
MR‐Lasso	0.008	0.007	0.025	0.086	0.033	0.024	0.054	0.267	0.123	0.089	0.130	0.597
MR‐Egger	0.092	0.074	0.234	0.105	0.099	0.090	0.277	0.091	0.094	0.089	0.312	0.084
Contamination mixture	0.000	0.001	0.020	0.062	0.002	0.002	0.029	0.093	0.026	0.005	0.156	0.166
MR‐Mix	0.029	0.010	0.141	0.095	0.056	0.010	0.220	0.139	0.125	0.020	0.327	0.154
MR‐RAPS	0.082	0.075	0.068	0.174	0.172	0.165	0.103	0.415	0.256	0.251	0.124	0.591

	Positive causal effect: θ=+0.2											
	30% invalid				50% invalid				70% invalid			
Method	Mean	Median	*SD*	Power	Mean	Median	*SD*	Power	Mean	Median	*SD*	Power
Scenario 1: Balanced pleiotropy, InSIDE satisfied								
Weighted mMedian	0.200	0.200	0.035	0.998	0.201	0.200	0.066	0.978	0.202	0.202	0.135	0.908
Mode‐based estimation	0.199	0.199	0.032	0.997	0.197	0.198	0.062	0.982	0.187	0.193	0.143	0.870
MR‐PRESSO	0.199	0.200	0.050	0.983	0.200	0.200	0.089	0.928	0.202	0.202	0.142	0.846
MR‐Robust	0.200	0.200	0.025	0.997	0.200	0.199	0.077	0.668	0.203	0.204	0.170	0.271
MR‐Lasso	0.200	0.200	0.026	1.000	0.200	0.200	0.038	0.996	0.201	0.201	0.080	0.942
MR‐Egger	0.200	0.199	0.311	0.149	0.209	0.211	0.396	0.120	0.196	0.196	0.462	0.102
Contamination mixture	0.201	0.201	0.023	1.000	0.201	0.200	0.032	0.997	0.215	0.203	0.194	0.943
MR‐Mix	0.209	0.200	0.141	0.606	0.211	0.200	0.233	0.793	0.182	0.170	0.353	0.200
MR‐RAPS	0.199	0.199	0.075	0.644	0.201	0.202	0.131	0.345	0.202	0.204	0.177	0.231
Scenario 2: Directional pleiotropy, InSIDE satisfied												
Weighted median	0.212	0.210	0.033	1.000	0.232	0.222	0.065	0.998	0.289	0.255	0.132	0.989
Mode‐based estimation	0.200	0.199	0.031	0.998	0.205	0.203	0.052	0.989	0.236	0.224	0.116	0.950
MR‐PRESSO	0.223	0.216	0.042	1.000	0.267	0.247	0.083	0.999	0.344	0.319	0.134	0.995
MR‐Robust	0.203	0.203	0.025	0.999	0.237	0.229	0.070	0.821	0.353	0.344	0.135	0.731
MR‐Lasso	0.204	0.204	0.025	1.000	0.216	0.213	0.041	1.000	0.266	0.244	0.101	0.994
MR‐Egger	0.202	0.194	0.222	0.217	0.197	0.188	0.277	0.150	0.204	0.197	0.331	0.126
Contamination mixture	0.201	0.201	0.022	1.000	0.203	0.203	0.034	0.999	0.234	0.206	0.193	0.969
MR‐Mix	0.230	0.210	0.141	0.461	0.263	0.220	0.232	0.518	0.328	0.230	0.378	0.502
MR‐RAPS	0.248	0.244	0.059	0.969	0.307	0.303	0.099	0.881	0.381	0.376	0.131	0.837
Scenario 3: Directional pleiotropy, InSIDE violated								
Weighted median	0.225	0.219	0.045	1.000	0.270	0.244	0.096	1.000	0.361	0.320	0.158	0.998
Mode‐based estimation	0.202	0.201	0.039	0.995	0.215	0.206	0.072	0.986	0.270	0.245	0.137	0.963
MR‐PRESSO	0.247	0.234	0.058	1.000	0.326	0.302	0.108	1.000	0.429	0.415	0.146	0.999
MR‐Robust	0.206	0.205	0.026	0.997	0.265	0.251	0.084	0.781	0.427	0.419	0.137	0.838
MR‐Lasso	0.209	0.208	0.027	1.000	0.235	0.226	0.056	1.000	0.326	0.293	0.131	0.999
MR‐Egger	0.289	0.269	0.231	0.316	0.305	0.295	0.276	0.250	0.297	0.293	0.314	0.201
Contamination mixture	0.201	0.202	0.023	1.000	0.204	0.203	0.036	0.999	0.248	0.209	0.208	0.974
MR‐Mix	0.241	0.215	0.150	0.505	0.288	0.223	0.248	0.593	0.362	0.250	0.366	0.546
MR‐RAPS	0.281	0.274	0.068	0.990	0.371	0.365	0.102	0.976	0.459	0.454	0.125	0.974

Abbreviations: InSIDE, Instrument Strength Independent of Direct Effect; MR, Mendelian randomization; PRESSO, Pleiotropy Residual Sum and Outlier; RAPS, Robust Adjusted Profile Score; SD, standard deviation.

**Table 4:** Mean, median, *SD* of estimates, and Type 1 error/empirical power (%) with 100 genetic variants

	Null casual effect: θ = 0											
	30% invalid				50% invalid				70% invalid			
Method	Mean	Median	*SD*	Type 1 error	Mean	Median	*SD*	Type 1 error	Mean	Median	*SD*	Type 1 error
Scenario 1: Balanced pleiotropy, InSIDE satisfied												
Weighted median	0.000	0.000	0.025	0.069	−0.001	0.000	0.041	0.124	0.000	0.000	0.077	0.234
Mode‐based estimation	0.000	0.000	0.024	0.038	0.000	0.000	0.035	0.082	0.000	0.000	0.084	0.333
MR‐PRESSO	0.000	0.000	0.025	0.134	0.000	0.001	0.047	0.224	0.000	−0.001	0.083	0.313
MR‐Robust	0.000	0.000	0.020	0.052	0.000	0.001	0.053	0.024	0.000	−0.001	0.126	0.044
MR‐Lasso	0.000	0.000	0.019	0.042	0.000	0.000	0.029	0.072	0.000	0.000	0.055	0.120
MR‐Egger	−0.001	−0.001	0.195	0.067	−0.001	0.000	0.252	0.069	−0.003	−0.005	0.296	0.065
Contamination mixture	0.000	0.000	0.019	0.064	0.000	0.000	0.029	0.088	0.002	0.000	0.211	0.136
MR‐Mix	0.000	0.000	0.075	0.038	−0.001	0.000	0.072	0.024	0.000	0.000	0.058	0.000
MR‐RAPS	0.000	−0.001	0.053	0.016	−0.001	0.000	0.095	0.036	0.000	−0.003	0.133	0.052
Scenario 2: Directional pleiotropy, InSIDE satisfied												
Weighted median	0.013	0.012	0.023	0.105	0.033	0.029	0.039	0.258	0.087	0.071	0.084	0.537
Mode‐based estimation	0.000	0.000	0.020	0.037	0.004	0.003	0.030	0.089	0.034	0.030	0.067	0.351
MR‐PRESSO	0.022	0.018	0.026	0.294	0.071	0.062	0.056	0.628	0.162	0.150	0.096	0.856
MR‐Robust	0.004	0.004	0.018	0.051	0.042	0.038	0.047	0.040	0.193	0.189	0.100	0.425
MR‐Lasso	0.004	0.004	0.017	0.077	0.020	0.018	0.029	0.242	0.076	0.066	0.067	0.617
MR‐Egger	0.001	−0.003	0.143	0.062	−0.002	−0.005	0.180	0.059	0.003	0.001	0.210	0.058
Contamination mixture	0.000	0.001	0.017	0.061	0.001	0.001	0.025	0.090	0.018	0.005	0.160	0.156
MR‐Mix	0.005	0.000	0.074	0.034	0.004	0.000	0.072	0.035	0.006	0.000	0.070	0.007
MR‐RAPS	0.058	0.056	0.042	0.142	0.140	0.138	0.072	0.435	0.233	0.232	0.097	0.663
Scenario 3: Directional pleiotropy, InSIDE violated								
Weighted median	0.027	0.025	0.027	0.258	0.077	0.065	0.062	0.619	0.184	0.163	0.116	0.881
Mode‐based estimation	0.001	0.001	0.021	0.042	0.010	0.008	0.035	0.120	0.065	0.054	0.087	0.465
MR‐PRESSO	0.053	0.047	0.040	0.658	0.152	0.142	0.079	0.943	0.276	0.270	0.103	0.993
MR‐Robust	0.007	0.007	0.019	0.054	0.078	0.071	0.059	0.080	0.292	0.289	0.099	0.805
MR‐Lasso	0.010	0.009	0.018	0.153	0.049	0.043	0.041	0.575	0.165	0.151	0.097	0.927
MR‐Egger	0.119	0.114	0.148	0.162	0.141	0.138	0.178	0.159	0.123	0.124	0.194	0.114
Contamination mixture	0.001	0.001	0.017	0.069	0.003	0.003	0.026	0.107	0.021	0.009	0.135	0.199
MR‐Mix	0.007	0.000	0.073	0.037	0.008	0.000	0.070	0.034	0.007	0.000	0.068	0.006
MR‐RAPS	0.104	0.101	0.049	0.545	0.224	0.221	0.076	0.896	0.330	0.327	0.090	0.976

	Positive causal effect: θ=+0.2											
	30% invalid				50% invalid				70% invalid			
Method	Mean	Median	SD	Power	Mean	Median	SD	Power	Mean	Median	SD	Power
Scenario 1: Balanced pleiotropy, InSIDE satisfied								
Weighted median	0.200	0.200	0.028	1.000	0.201	0.201	0.043	0.996	0.201	0.200	0.078	0.939
Mode‐based estimation	0.199	0.199	0.025	1.000	0.199	0.199	0.036	0.998	0.192	0.192	0.085	0.908
MR‐PRESSO	0.200	0.200	0.026	1.000	0.201	0.200	0.047	0.993	0.201	0.200	0.083	0.934
MR‐Robust	0.200	0.200	0.021	1.000	0.201	0.202	0.055	0.896	0.200	0.200	0.126	0.373
MR‐Lasso	0.200	0.200	0.020	1.000	0.201	0.201	0.031	1.000	0.200	0.200	0.057	0.986
MR‐Egger	0.200	0.200	0.199	0.212	0.199	0.200	0.248	0.146	0.206	0.206	0.298	0.130
Contamination mixture	0.202	0.202	0.021	1.000	0.203	0.204	0.031	1.000	0.228	0.206	0.253	0.977
MR‐Mix	0.203	0.200	0.091	0.979	0.191	0.200	0.105	0.873	0.028	0.000	0.103	0.001
MR‐RAPS	0.201	0.201	0.054	0.880	0.201	0.199	0.095	0.504	0.201	0.202	0.133	0.332
Scenario 2: Directional pleiotropy, InSIDE satisfied												
Weighted median	0.214	0.213	0.025	1.000	0.237	0.233	0.043	1.000	0.290	0.275	0.086	1.000
Mode‐based estimation	0.199	0.199	0.023	1.000	0.203	0.203	0.033	1.000	0.229	0.226	0.071	0.986
MR‐PRESSO	0.222	0.219	0.027	1.000	0.271	0.263	0.056	1.000	0.362	0.351	0.095	1.000
MR‐Robust	0.205	0.205	0.020	1.000	0.247	0.243	0.051	0.995	0.395	0.390	0.100	0.982
MR‐Lasso	0.205	0.205	0.019	1.000	0.223	0.220	0.032	1.000	0.281	0.270	0.071	1.000
MR‐Egger	0.201	0.198	0.144	0.325	0.203	0.199	0.182	0.229	0.201	0.199	0.213	0.187
Contamination mixture	0.202	0.202	0.019	1.000	0.204	0.204	0.028	1.000	0.256	0.211	0.271	0.995
MR‐Mix	0.208	0.200	0.094	0.641	0.211	0.200	0.098	0.899	0.055	0.000	0.136	0.061
MR‐RAPS	0.260	0.257	0.043	1.000	0.342	0.340	0.073	0.997	0.434	0.431	0.098	0.994
Scenario 3: Directional pleiotropy, InSIDE violated								
Weighted median	0.230	0.228	0.029	1.000	0.282	0.271	0.065	1.000	0.389	0.369	0.116	1.000
Mode‐based estimation	0.201	0.200	0.024	1.000	0.210	0.208	0.039	0.999	0.263	0.252	0.088	0.988
MR‐PRESSO	0.252	0.246	0.039	1.000	0.349	0.339	0.077	1.000	0.474	0.468	0.100	1.000
MR‐Robust	0.209	0.209	0.022	1.000	0.287	0.280	0.063	0.986	0.495	0.492	0.097	0.999
MR‐Lasso	0.212	0.212	0.021	1.000	0.254	0.248	0.045	1.000	0.372	0.359	0.096	1.000
MR‐Egger	0.321	0.314	0.146	0.640	0.343	0.339	0.180	0.534	0.327	0.323	0.194	0.420
Contamination mixture	0.203	0.203	0.020	1.000	0.206	0.205	0.031	1.000	0.268	0.217	0.269	0.995
MR‐Mix	0.211	0.205	0.094	0.730	0.211	0.200	0.101	0.900	0.058	0.000	0.150	0.033
MR‐RAPS	0.306	0.303	0.050	1.000	0.426	0.424	0.077	1.000	0.531	0.529	0.088	1.000

Abbreviations: InSIDE, Instrument Strength Independent of Direct Effect; MR, Mendelian randomization; PRESSO, Pleiotropy Residual Sum and Outlier; RAPS, Robust Adjusted Profile Score; SD, standard deviation.

Overall, judging by mean squared error, the contamination mixture method performed best with 30% and 50% invalid variants. In some scenarios, other methods had lower mean squared error with 70% invalid variants. However, with some isolated exceptions, all the methods performed badly with 70% invalid instruments. Coverage for the contamination mixture method was around 10% or less when there were up to 50% invalid variants. This was also true for the MR‐Robust method, although that method had slightly lower power to detect a causal effect in some scenarios. Several other methods performed well in particular scenarios.

Among consensus methods, estimates from the MBE method were less biased than those from the weighted median method, with lower Type 1 errors. The weighted median method had slightly higher power to detect a causal effect, although comparisons of power lose much of their value when a method has inflated Type 1 error rates. Performance of the MBE method improved as the number of variants increased. Among outlier‐robust methods, bias was greater for the MR‐Robust than the MR‐Lasso method. The MR‐Lasso method generally had the lower mean squared error when the invalidity was 50% or 70%, but MR‐Robust had the lower Type 1 error rates. Performance of the MR‐Robust method was better when there were at least 30 genetic variants. MR‐PRESSO had biased estimates with inflated Type 1 error rates even with 30% invalid variants, and performed particularly badly as the number of variants increased.

The modelling methods performed well in some scenarios, but less well in others. This is unsurprising, as in some scenarios, consistency assumptions for the methods were satisfied, and in others they were not. The MR‐Egger method performed well in terms of Type 1 error rate in Scenarios 1 and 2, where the InSIDE assumption was satisfied. Estimates from the method were generally imprecise with low power. However, power in the MR‐Egger method depends on the genetic associations with the exposure varying substantially between variants, which was not the case in the simulation study (Burgess & Thompson, 2017). The contamination mixture method performed well with 30% and 50% valid instruments, with low bias and Type 1 error rates at or below 8% with 10 variants, 10% with 30 variants, and 11% with 100 variants. The MR‐Mix method performed badly throughout, with highly inflated Type 1 error rates in almost all scenarios with less than 100 instruments and comparatively low power to detect a causal effect. It performed slightly better with more genetic variants, although its performance was still worse than other methods. However, the method performed much better in a simulation comparison of methods performed by the authors of the MR‐Mix method (Qi & Chatterjee, 2019), in which the data‐generating model was more similar to the model assumed by the MR‐Mix method. The MR‐RAPS method performed well in Scenario 1, where its consistency assumption was satisfied, but less well in other scenarios with inflated Type 1 error rates. Its performance also worsened as more variants were included in the analysis.

### 3.2 Empirical example: The effect of BMI on coronary artery disease

Results from the empirical example are shown in Table 5. All methods agree that there is a positive effect of BMI on CAD risk, except for the MR‐Mix method which gives a wide confidence interval that includes the null. The narrowest confidence intervals are for the outlier‐robust methods (MR‐Lasso, MR‐Robust, MR‐PRESSO), followed by the modelling methods except MR‐Mix and MR‐Egger (contamination mixture, MR‐RAPS), then the consensus methods (weighted median, MBE), and finally MR‐Egger and MR‐Mix.

**Table 5:** Estimates and 95% CI for the effect of BMI on coronary artery disease risk from robust methods

Method	Causal estimate (95% CI)	CI width
Weighted median	0.376 (0.206, 0.546)	0.340
Mode‐based estimation	0.382 (0.181, 0.583)	0.402
MR‐PRESSO	0.410 (0.309, 0.511)	0.202
MR‐Robust	0.425 (0.325, 0.526)	0.201
MR‐Lasso	0.442 (0.354, 0.530)	0.176
MR‐Egger	0.481 (0.165, 0.796)	0.631
(intercept)	−0.003 (−0.011, 0.005)	
Contamination mixture	0.490 (0.372, 0.602)	0.230
MR‐Mix	0.425 (−0.283, 1.133)	1.416
MR‐RAPS	0.390 (0.308, 0.546)	0.238

*Note*: Estimates represent log odds ratios for CAD risk per 1 kg/m^2^ increase in BMI.

Abbreviations: BMI, body mass index; CI, confidence intervals; PRESSO, Pleiotropy Residual Sum and Outlier; RAPS, Robust Adjusted Profile Score.

While the methods that detect outliers varied in terms of how lenient or strictly they identified outliers, they agreed on the order of outliers (Table S3). The MR‐Robust method was the most lenient, downweighting two variants as outliers. Each subsequent method in order of strictness identified all previously identified variants as outliers. MR‐PRESSO excluded the two variants identified by MR‐Robust plus an additional three variants. MR‐RAPS identified these five plus an additional two variants. MR‐Lasso identified an additional three variants, 10 in total. The contamination mixture method identified an additional 14 variants, 24 in total. MR‐Mix identified an additional 21 variants, 45 in total. This suggests that any difference between results from outlier‐robust methods are likely due to the strictness of outlier detection, rather than due to intrinsic differences in how the different methods select outliers. In several methods, the threshold at which outliers are detected can be varied by the analyst (e.g., by varying the penalization parameter λ in MR‐Lasso, or the significance threshold in MR‐PRESSO). In practice, rather than performing different outlier‐robust methods, it may be better to concentrate on one method, but vary this threshold. In our example, some of the variants that were the most pleiotropic in terms of their associations with other measured risk factors were only removed from the analysis by the MR‐Mix method (Table S3).

## 4 DISCUSSION

In this paper, we have provided a review of robust methods for MR, focusing on methods that can be performed using summary data and implemented using standard statistical software. We have divided methods into three categories: consensus methods, outlier‐robust methods, and modelling methods. Methods were compared in three ways: by their theoretical properties, including the assumptions required for the method to give a consistent estimate, in an extensive simulation study, and in an empirical investigation.

While the use of robust methods for MR analyses with multiple genetic variants is highly recommended, it is not practical or desirable to perform and report results from every single robust method that has been proposed. Guidance is therefore needed as to which robust methods should be performed in practice. As an example, if an investigator performed the MR‐PRESSO, MR‐Robust, and MR‐Lasso methods, they would have assessed robustness of the result to outliers, but they would not have not assessed other potential violations of the IV assumptions. The categorization of methods proposed here is not the only possible division of methods, but we hope it is practically useful. For instance, the contamination mixture and MR‐Mix methods make the same “plurality valid” assumption as the MBE method, and so could have been placed in the same category.

The similarity and ubiquity of the “outlier‐robust” and “majority/plurality valid” assumptions should encourage investigators to consider methods that make alternative assumptions, such as the MR‐Egger method. While the InSIDE assumption is often not plausible (Burgess & Thompson, 2017), the MR‐Egger method and the intercept test have value in providing a different route to testing the validity of an MR study. Another potential choice is the constrained IV method, which uses information on measured confounders to construct a composite IV that is not associated with these confounders (Jiang et al., 2017). This method was not considered in the simulation study, as it requires additional data on confounders and individual participant data. Further methods development is needed to develop robust methods for summary data that make different consistency assumptions.

We encourage researchers to perform robust methods from different categories, and that make varied consistency assumptions. For example, an investigator could perform the weighted median method (majority valid assumption), the contamination mixture method (plurality valid assumption), and the MR‐Egger method (InSIDE assumption). If there are a few clear outliers in the data, then an outlier‐robust method such as MR‐PRESSO (best used with few very distinct outliers) or MR‐Robust could also be performed. While we are hesitant to make a definitive recommendation as each method has its own strengths and weaknesses, this set of methods would be a reasonable compromise between performing too few methods and not adequately assessing the IV assumptions, and performing so many methods that clarity is obscured. Another danger of the use of large numbers of methods is the possibility to cherry‐pick results, either by an investigator seeking to present their results in a more positive light, or a reader picking the one method that gives a different result (such as the MR‐Mix method in our empirical example).

One important limitation of these methods is the assumption that all valid IVs estimate the same causal effect. Particularly for complex exposures such as BMI, it is possible that different genetic variants have different ratio estimates not because they are invalid IVs, but because there are different ways of intervening on BMI that lead to different effects on the outcome. This can be remedied somewhat in methods based on the IVW method by using a random‐effects model (Bowden et al., 2017), or in the contamination mixture method, where causal effects evidenced by different sets of variants will lead to a multimodal likelihood function, and potentially a confidence interval that consists of more than one region.

In summary, while robust methods for MR do not provide a perfect solution to violations of the IV assumptions, they are able to detect such violations and help investigators make more reliable causal inferences. Investigators should perform a range of robust methods that operate in different ways and make different assumptions to assess the robustness of findings from a MR investigation.

## Supporting information

Supplementary Information

## Data Availability

The applied data that support the findings of this study are already available through PhenoScanner (amongst others). The simulated data is not available.
