# Supplementary material for: A comparison of robust Mendelian randomization methods using summary data
Source: Genet Epidemiol. 2020 Apr 6;44(4):313–29. doi: 10.1002/gepi.22295 (PMC7317850; doi:10.1002/gepi.22295)
Supplement: Supplementary file 1 — Supplementary Information [file GEPI-44-313-s001.pdf]

Appendix

Eric A.W. Slob[1,2,*] and Stephen Burgess[3]

[1]Erasmus School of Economics, Erasmus University Rotterdam, Rotterdam, The Netherlands

[2]Erasmus University Rotterdam Institute for Behavior and Biology, Rotterdam, The Netherlands

[3]Department of Public Health and Primary Care, University of Cambridge, Cambridge, United Kingdom

*Corresponding E-mail: e.a.w.slob@ese.eur.nl. Telephone: +31 10 4088946.

Supporting Information

S1 Details of simulation study

For each participant i, we simulate data on J genetic variants $G_{i1}, G_{i2}, \ldots, G_{iJ}$, a modifiable exposure X_i, an outcome variable Y_i, and a confounder U_i (assumed unknown). The confounder is a linear function of the genetic variants and an independent error term ε_i^U. The effect of variant j on the confounder is represented by coefficient ϕ_j (this is zero for a valid IV). The exposure is linear in the genetic variants, the confounder and an independent error term ε_i^X. The effect of variant j on the exposure is represented by coefficient γ_j. The outcome is linear in the genetic variants, exposure, confounders and an independent error term ε_i^Y. The effect of variant j on the outcome is represented by coefficient α_j (again, this is zero for a valid IV). The effect of the exposure on the outcome is represented by θ. The genetic variants are modelled as single nucleotide polymorphisms (SNPs), with a varying minor allele frequency maf$_j$, and take values 0, 1 or 2. The minor allele frequencies are drawn from an uniform distribution (maf$_j \sim \mathcal{U}(0.1, 0.5)$). The error terms ε_i^U, ε_i^X and ε_i^Y each follow an independent normal distribution with mean 0 and unit variance.

We can represent the model mathematically as:

$$U_i = \sum_{j=1}^{J} \phi_j G_{ij} + \varepsilon_i^U, \quad (1)$$

$$X_i = \sum_{j=1}^{J} \gamma_j G_{ij} + U_i + \varepsilon_i^X, \quad (2)$$

$$Y_i = \sum_{j=1}^{J} \alpha_j G_{ij} + \theta\, X_i + U_i + \varepsilon_i^Y, \quad (3)$$

$$\text{maf}_j \sim \mathcal{U}(0.1, 0.5), \quad (4)$$

$$G_{ij} \sim \text{Binomial}(2, \text{maf}_j) \text{ independently}, \quad (5)$$

$$\varepsilon_i^U, \varepsilon_i^X, \varepsilon_i^Y \sim \mathcal{N}(0, 1) \text{ independently}. \quad (6)$$

The causal effect of the exposure on the outcome was either taken as null ($\theta = 0$) or positive ($\theta = 0.2$). Genetic associations with the exposure γ_j are drawn from a left-sided truncated normal distribution (truncation at 0.15, 0.1, and 0.05, for $J = 10$, 30, and 100 respectively). The variance of this distribution is chosen such that the total proportion of variance explained in the exposure by direct effects of the genetic variants is on average 10%. In scenario 3, the overall proportion of variance explained in the exposure by genetic variants is slightly larger, as there is an additional effect of the invalid IVs on the exposure via their effect on the confounder.

For valid IVs, $\phi_j = 0$ and $\alpha_j = 0$. For invalid IVs, in scenario 1 (balanced pleiotropy, InSIDE satisfied), the effects of the genetic variants on the outcome are generated from a normal distribution centered at zero ($\alpha_j \sim \mathcal{N}(0, 0.15)$) and genetic effects on the confounder are zero ($\phi_j = 0$). In scenario 2 (directional pleiotropy, InSIDE satisfied), the effects of the genetic variants on the outcome are generated from a normal distribution centered away from zero ($\alpha_j \sim \mathcal{N}(0.1, 0.075)$) and genetic effects on the confounder are zero ($\phi_j = 0$). In scenario 3 (directional pleiotropy, InSIDE violated), the direct effects of the genetic variants on the outcome are generated from a normal distribution centered away from zero ($\alpha_j \sim \mathcal{N}(0.1, 0.075)$) and genetic effects on the confounder are generated from a uniform distribution ($\phi_j \sim \mathcal{U}(0, 0.1)$).

Summary genetic association data are calculated by regressing the outcome on each genetic variant in turn. Individual participant data are generated for 10 000 individuals, where we perform the outcome regressions on all these individuals to come to the second stage effect estimates and corresponding standard errors. For the exposure summary genetic associations, we give the true value of the first stage effect with corresponding theoretical standard error (which is given by $(\sqrt{N} * \sqrt{2 * \text{maf}_j * (1 - \text{maf}_j)})^{-1}$, where N is the number of individuals in the first stage GWAS) with again 10 000 individuals. This represents a two-sample Mendelian randomization study. We generated 10 000 simulated datasets for each scenario, and for null and positive causal effects.

Each method is performed using the default options suggested by the authors of the method, either in the corresponding publication, or in the software code recommended by the authors. The weighted median method is performed using inverse-variance weights. The mode based estimation method is

performed using inverse-variance weights, the 'no measurement error' assumption, and the default bandwidth setting ($\phi = 1$). The MR-PRESSO method is performed using a significance cut-off of $p < 0.05$ for determining outliers. The MR-Lasso method is performed using the heterogeneity criterion for selecting the lasso penalty parameter. The contamination mixture method is performed using the standard deviation of the ratio estimates multiplied by 1.5 for the variance parameter. For MR-Mix, we choose an initial value of the probability mass at the null component as 0.6 and the initial value of the variance of the non-null component as 1×10^{-5}. As the method performs a grid search, these decisions should not influence the results. For MR-RAPS, we use the overdispersed robust version with the Huber loss function. All regression models use random-effects.

The mean squared errors of the different methods are presented in Supplementary Figure 1 (10 variants, scenario 2), Supplementary Figure 2 (10 variants, scenario 3), Supplementary Figure 3 (100 variants, scenario 2), and Supplementary Figure 4 (100 variants, scenario 3). Note that in each case the vertical axis is on a logarithmic scale. Findings are similar to before among the different scenarios. We observe again that the performance of the mode based estimator is the best for the consensus based approach, MR-Robust gets the best result among the outlier-robust methods, and the contamination mixture approach has the best performance among the modelling methods.

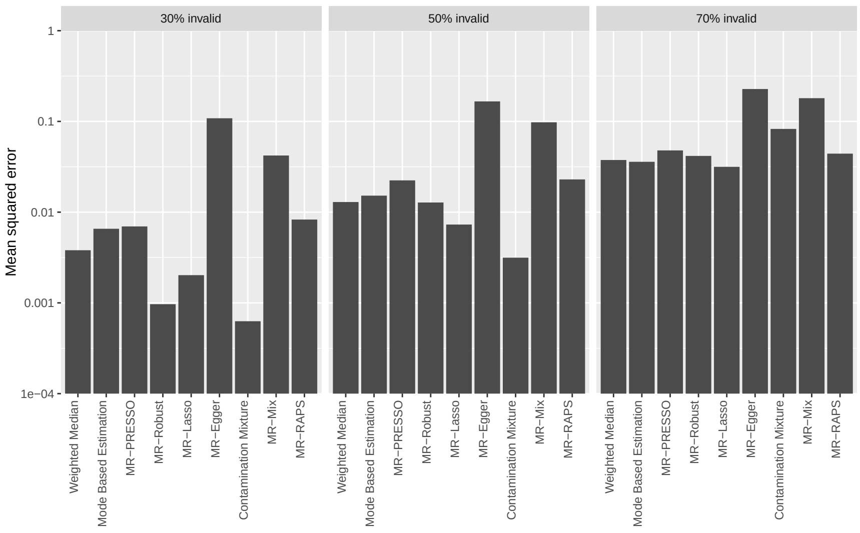

Fig. 1 Mean squared error for the different methods in scenario 2 for 10 000 simulations, with directional pleiotropy and InSIDE satisfied with 10 variants.

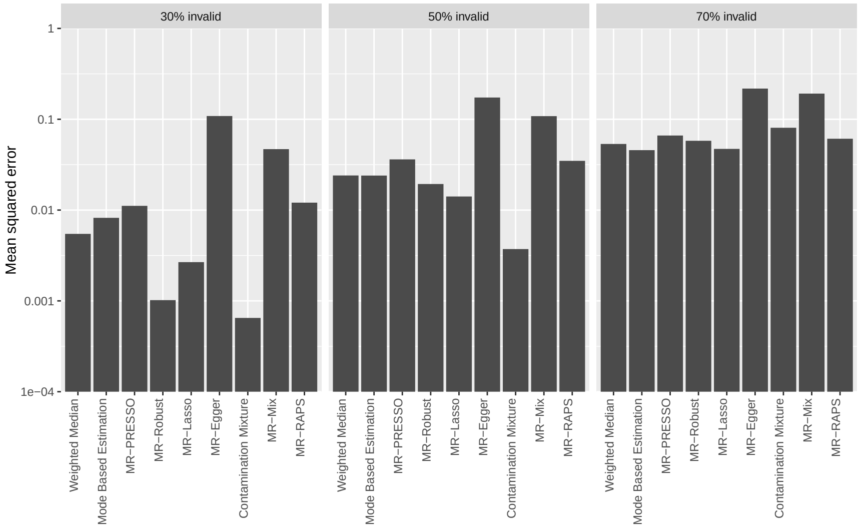

Fig. 2 Mean squared error for the different methods in scenario 3 for 10 000 simulations, with directional pleiotropy and InSIDE violated with 10 variants.

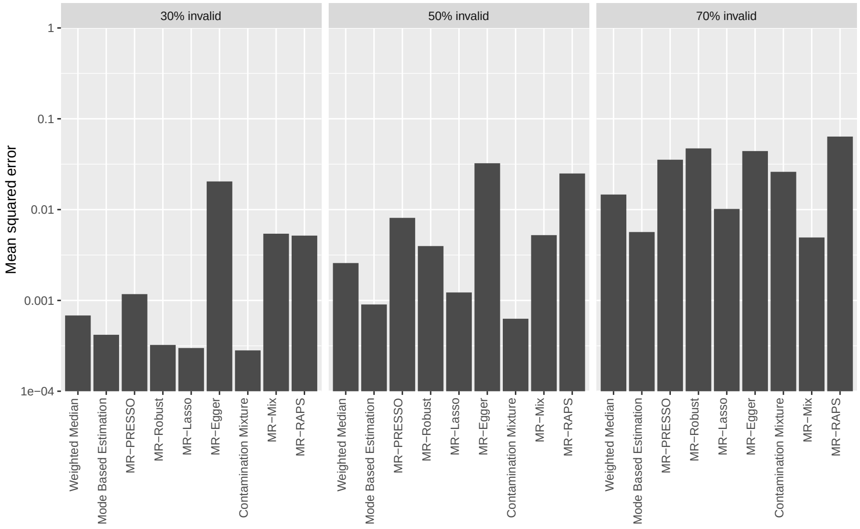

Fig. 3 Mean squared error for the different methods in scenario 2 for 10 000 simulations, with directional pleiotropy and InSIDE satisfied with 100 variants.

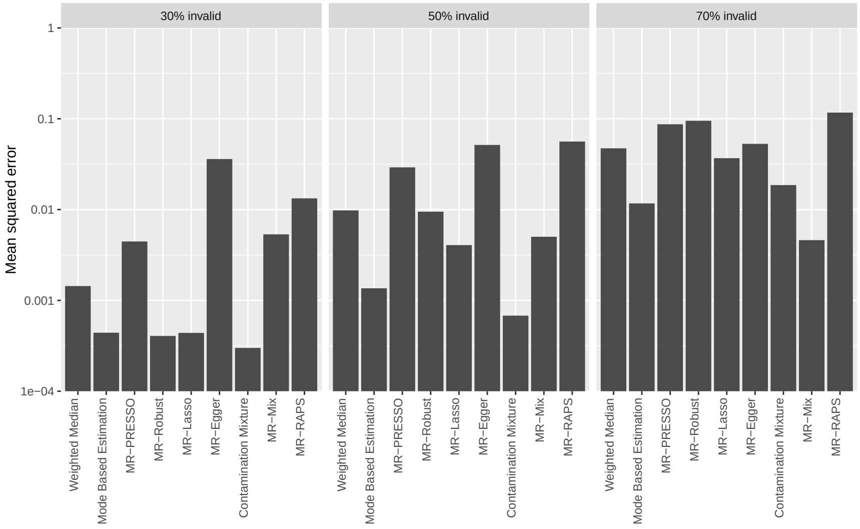

Fig. 4 Mean squared error for the different methods in scenario 3 for 10 000 simulations, with directional pleiotropy and InSIDE violated with 100 variants.

S2 Outliers according to different methods

Table 1 Genetic variants identified as outliers by the different methods in the Mendelian Randomization study of the effect of BMI on cardiovascular disease risk and other traits the variants are associated with according to the NHGRI-EBI Catalog of published genome-wide association studies (Buniello et al., 2019) (last accessed on 12 July 2019).

Variant	MR-Robust	MR-PRESSO	MR-RAPS	MR-Lasso	Contamination mixture	MR-Mix	Associated traits
rs11191560	✓	✓	✓	✓	✓	✓	
rs2075650	✓	✓	✓	✓	✓	✓	Waist circumference, Hip circumference
rs2176040		✓	✓	✓	✓	✓	Waist circumference, Obesity
rs6567160		✓	✓	✓	✓	✓	Hand grip strength
rs7903146		✓	✓	✓	✓	✓	Hip circumference
rs11727676			✓	✓	✓	✓	Parental longevity
rs17024393			✓	✓	✓	✓	
rs11126666				✓	✓	✓	
rs13078960				✓	✓	✓	HDL cholesterol levels, Colorectal cancer, Diverticular disease
rs9914578				✓	✓	✓	
rs1000940					✓	✓	
rs11057405					✓	✓	Waist circumference, Hip circumference
rs11847697					✓	✓	
rs12446632					✓	✓	
rs12566985					✓	✓	
rs16907751					✓	✓	
rs205262					✓	✓	
rs2650492					✓	✓	
rs2836754					✓	✓	Hip circumference, Waist circumference, Obesity
rs3849570					✓	✓	
rs4787491					✓	✓	
rs492400					✓	✓	Hip circumference, Hand grip strength
rs7243357					✓	✓	
rs9641123					✓	✓	
rs10938397						✓	Alzheimer's disease, C-reactive protein, Age-related macular degeneration, Cerebrospinal fluid levels, Waist-hip ratio, Waist circumference, Longevity, LDL cholesterol, Total cholesterol, Cognitive decline, Cognitive impairment score, Cerebral amyloid deposition
rs10968576						✓	Waist Circumference
rs11030104						✓	
rs11688816						✓	Waist circumference, Waist-hip ratio
rs12016871						✓	Waist circumference, Intelligence
rs13021737						✓	Waist circumference
rs13191362						✓	Crohn's disease
rs13201877						✓	Waist circumference, Fat-free mass
rs1460676						✓	Intelligence
rs1516725						✓	Waist circumference
rs1528435						✓	Feeling nervous
rs17203016						✓	
rs2176598						✓	Body fat percentage, Hip circumference
rs2287019						✓	Body fat percentage, Waist circumference, Fat-free mass
rs2820292						✓	
rs3810291						✓	
rs3817334						✓	
rs543874						✓	
rs7164727						✓	Type 2 diabetes, Hip circumference, Waist circumference, Peak insulin response, Clinical laboratory measurements, Metabolic syndrome, Glycated hemoglobin levels, Schizophrenia, Systolic blood pressure, Pulse pressure, Fasting blood insulin, Fasting blood glucose, Proinsulin levels
rs7599312						✓	Lean body mass
rs7899106						✓	

S3 Software code

This section includes the code to run the robust methods used in this paper. Please note that the MR-Mix package is not publicly available, please contact the authors for the package [1].

```
#install required packages
if (!require("MendelianRandomization")) {install.packages("
   MendelianRandomization")} else {}
if (!require("mr.raps")) {install.packages("mr.raps")} else {}
if (!require("devtools")) { install.packages("devtools") } else {}
if (!require("penalized")) {install.packages("penalized")} else {}

library("devtools")
devtools::install_github("rondolab/MR-PRESSO")

#load packages
library("MendelianRandomization")
library("mr.raps")
library("MRMix")
library("MRPRESSO")
library("penalized")

#create dataframe and object by different methods
mr_frame<-as.data.frame(cbind(ldlc,ldlcse,chdlodds,chdloddsse))
names(mr_frame)<-c("ldlc","ldlcse","chdlodds","chdloddsse")
mr_object<-mr_input(bx = ldlc, bxse = ldlcse, by = chdlodds, byse =
     chdloddsse)#create used by methods from MendelianRandomization
   package

#perform weighted median
mr_median(mr_object,weighting = "weighted", iterations = 10000)

#perform Mode based estimation
mr_mbe(mr_object, weighting = "weighted", stderror = "delta", phi =
     1,
      seed = 19940407, iterations = 10000, distribution = "normal",
      alpha = 0.05)
#perform MR-PRESSO
mr_presso(BetaOutcome = "chdlodds", BetaExposure = "ldlc",
   SdOutcome = "chdloddsse", SdExposure = "ldlcse", OUTLIERtest =
```

[1] current maintainer of the package is Guanghao Qi (gqi1@jhu.edu).

```
    TRUE, DISTORTIONtest = TRUE, data = mr_frame, NbDistribution =
    1000, SignifThreshold = 0.05)



#perform MR-Robust
mr_ivw(mr_object,"random", robust = TRUE)

#define function for MR-Lasso with heterogeneity criterion
MR_lasso<-function(betaYG,betaXG,sebetaYG){

  betaYGw = betaYG/sebetaYG # dividing the association estimates by
      sebetaYG is equivalent
  betaXGw = betaXG/sebetaYG # to weighting by sebetaYG^-2
  pleio = diag(rep(1, length(betaXG)))
  l1grid = c(seq(from=0.1, to=5, by=0.1), seq(from=5.2, to=10, by
      =0.2))
  # values of lambda for grid search
  l1grid_rse = NULL; l1grid_length = NULL; l1grid_beta = NULL;
      l1grid_se = NULL
  for (i in 1:length(l1grid)) {
    l1grid_which = which(attributes(penalized(betaYGw, pleio,
       betaXGw, lambda1=l1grid[i], trace=FALSE))$penalized==0)
    l1grid_rse[i] = summary(lm(betaYG[l1grid_which]~betaXG[l1grid_
       which]-1, weights=sebetaYG[l1grid_which]^-2))$sigma
    l1grid_length[i] = length(l1grid_which)
    l1grid_beta[i] = lm(betaYG[l1grid_which]~betaXG[l1grid_which]-1,
        weights=sebetaYG[l1grid_which]^-2)$coef[1]
    l1grid_se[i] = summary(lm(betaYG[l1grid_which]~betaXG[l1grid_
       which]-1, weights=sebetaYG[l1grid_which]^-2))$coef[1,2]/min(
       summary(lm(betaYG[l1grid_which]~betaXG[l1grid_which]-1,
       weights=sebetaYG[l1grid_which]^-2))$sigma, 1)
  }
  l1which_hetero = c(which(l1grid_rse[1:(length(l1grid)-1)]>1& diff(
      l1grid_rse)>qchisq(0.95, df=1)/l1grid_length[2:length(l1grid)])
      , length(l1grid))[1]
  # heterogeneity criterion for choosing lambda

  l1hetero_beta = l1grid_beta[l1which_hetero]
  l1hetero_se = l1grid_se[l1which_hetero]
```

```
  list(ThetaEstimate=l1hetero_beta, ThetaSE=l1hetero_se )

}





#perform MR-Lasso
MR_lasso(mr_frame$chdlodds,mr_frame$ldlc,mr_frame$chdloddsse)

#perform MR-Egger
mr_egger(mr_object)

#define function for contamination mixture
contaminationmixture<-function(by,bx,byse){
  iters = 2001; theta = seq(from=-3, to=3, by=2/(iters-1))
  # if the causal estimate (and confidence interval) is not expected
      to lie between -1 and 1 then change from and to (and maybe
     increase iters)
  ratio = by/bx; ratio.se = abs(byse/bx); psi = 1.5*sd(ratio)
  lik=NULL
  for (j1 in 1:iters) {
   lik.inc = exp(-(theta[j1]-ratio)^2/2/ratio.se^2) /sqrt(2*pi*
      ratio.se^2)
   lik.exc = exp(-ratio^2/2/(psi^2+ratio.se^2)) /(sqrt(2*pi*(psi^2+
      ratio.se^2)))
   valid = (lik.inc>lik.exc)*1
   lik[j1] = prod(c(lik.inc[valid==1], lik.exc[valid==0]))
   if (which.max(lik)==length(lik)) { valid.best = valid }
  }
  phi = ifelse(sum(valid.best)<1.5, 1, max(sqrt(sum(((ratio[valid.
     best==1]-weighted.mean(ratio[valid.best==1] , ratio.se[valid.
     best==1]^-2))^2 * ratio.se[valid.best==1]^-2)) /(sum(valid.best
     )-1)), 1))
  loglik = log(lik)
  whichin = which(2*loglik>(2*max(loglik)-qchisq(0.95, df=1)*phi^2))
  theta[which.max(loglik)] # estimate
  theta[whichin[1]] # lower limit of CI
  theta[whichin[length(whichin)]] # upper limit of CI
```

```
  list(ThetaEstimate=theta[which.max(loglik)], ThetaLower=theta[
      whichin[1]] , ThetaUpper= theta[whichin[length(whichin)]] )
}



#perform contamination mixture, note we removed the 27th variable
    due to having a ratio to close to infty.
contaminationmixture(mr_frame$chdlodds[-27],mr_frame$ldlc[-27],mr_
    frame$chdloddsse[-27])




#perform MR-Mix
estMix = MRMix(mr_frame$chdlodds, mr_frame$ldlc, mr_frame$
    chdloddsse^2, mr_frame$ldlcse^2)
se = MRMix_se(mr_frame$chdlodds, mr_frame$ldlc, mr_frame$chdloddsse
    ^2, mr_frame$ldlcse^2, estMix$theta, estMix$pi0, estMix$sigma2)

#perform MR-RAPS with Huber loss function

mr.raps.overdispersed.robust(mr_frame$chdlodds, mr_frame$ldlc, mr_
    frame$chdloddsse, mr_frame$ldlcse,
                          loss.function = "huber", k = 1.345,
                              initialization = c("l2"), suppress.warning
                               = FALSE, diagnosis = FALSE, niter = 20,
                              tol = .Machine$double.eps^0.5)
```

References

Buniello, A., MacArthur, J. A. L., Cerezo, M., et al. 2019. The NHGRI-EBI GWAS catalog of published genome-wide association studies, targeted arrays and summary statistics 2019. *Nucleic acids research*, 47(D1):D1005–D1012.
